# Distinct Functions of Specialized Dendritic Cell Subsets in Atherosclerosis and the Road Ahead

**DOI:** 10.1155/2014/952625

**Published:** 2014-04-10

**Authors:** Alma Zernecke

**Affiliations:** Institute of Clinical Biochemistry and Pathobiochemistry, University Hospital Würzburg, Josef-Schneider-Straße 2, 97080 Würzburg, Germany

## Abstract

Atherosclerotic vascular disease is modulated by immune mechanisms. Dendritic cells (DCs) and T cells are present within atherosclerotic lesions and function as central players in the initiation and modulation of adaptive immune responses. In previous years, we have studied the functional contribution of distinct DC subsets in disease development, namely, that of CCL17-expressing DCs as well as that of plasmacytoid DCs that play specialized roles in disease development. This review focuses on important findings gathered in these studies and dissects the multifaceted contribution of CCL17-expressing DCs and pDCs to the pathogenesis of atherosclerosis. Furthermore, an outlook on future challenges faced when studying DCs in this detrimental disease are provided, and hurdles that will need to be overcome in order to enable a better understanding of the contribution of DCs to atherogenesis are discussed, a prerequisite for their therapeutic targeting in atherosclerosis.

## 1. Introduction

Atherosclerotic vascular disease remains the number one cause of death and morbidity in the Western world [1, 2]. Initially triggered by an endothelial cell dysfunction and activation under different cardiovascular risk factors (including hyperlipidemia, hypertension, and diabetes), the continued adhesion of leukocytes to the endothelium and their recruitment to the vessel wall, together with an increased permeability for plasma lipid components such as low-density lipoprotein (LDL), subsequently promote lesion growth. Monocytes, which take up cell-activating oxidized LDL (oxLDL) and other lipids, transform into foam cells characteristic of early fatty-streak lesions in the intima. Continued growth of the lipid core, recruitment of inflammatory cells, secretion of cytokines and growth factors, apoptosis of plaque cells, and the formation of a necrotic core lead to the continued stenosis of the arterial vessel. Secretion of matrix proteases and cytokines can ultimately trigger thinning of the fibrous cap which covers the core, leading to plaque erosion or rupture, which can subsequently trigger acute thrombus formation and occlusion of the artery [1, 2]. Besides monocytes/macrophages, which constitute the largest cell populations within atherosclerotic plaques, also other mononuclear cells, namely, T cells and antigen-presenting dendritic cells (DCs), can be detected within atherosclerotic lesions, and it is now well established that atherosclerosis is modulated by immune mechanisms [3, 4].

DCs can be subdivided into different subsets that play specialized roles in priming of adaptive immune responses [5]. In mice, DCs are divided into conventional DCs (cDCs) (further subdivided based on their expression of CD8*α* and CD4), plasmacytoid DCs (pDCs), and inflammatory DCs, for example, GM-CSF-driven monocyte-derived DCs or disease-triggered TNF-*α* and iNOS-producing DCs that are not found in the steady-state [5–8]. CD11c is commonly accepted as a pan-DC marker also for vascular DCs [4, 9–13].

A network of CD11c^+^ DCs has been identified in the arterial intima of healthy young individuals, and DCs accumulate in atherosclerosis-susceptible regions in mice [14, 15]. In advanced human plaques increased numbers of DCs are found in clusters with T cells, and DC-derived chemokines, such as CCL17 (also known as TARC/*thymus- and activation-regulated chemokine*), can be detected in atherosclerotic lesions. Similarly, pDCs are found in human atherosclerotic lesions [2, 16–21]. As professional antigen-presenting cells, DCs are essential for priming of immune responses [5, 6]. Indeed, we and others have shown that vascular CD11c^+^ DCs sorted from the aorta bear the capacity to induce antigen-specific proliferation of T cells* in vitro* [22, 23]. The presence of antigen-specific and clonally expanded T cells with strong reactivity for modified or native lipoproteins in human plaques further indicates that interactions between DCs and T cells can result in immune priming or reencounter of antigen at these sites* in vivo* [3, 24, 25]. Notably, endogenous antigen presentation and T-cell activation seem to play an important role in atherosclerosis.* Ldlr* ^−/−^ mice lacking the invariant chain of MHC-II (CD74) are protected from atherogenesis, mediated by reduced T-cell activation in atheromata [26].

Despite this evidence suggesting a role of DCs in the pathogenesis of atherosclerosis, the precise role of distinct DC subsets and their effector functions remain to be elucidated. Attempts at depleting DCs for longer time periods in order to assess functions of DCs in disease development have been difficult to interpret. The transient depletion of DCs in mice carrying a transgene encoding a diphtheria toxin receptor under the control of the CD11c-promoter was shown to induce apoptosis in CD11c^+^ plaque-macrophages with subsequent proinflammatory effects on atherosclerosis [27], or prevailing effects of other cell subsets (Zernecke et al., unpublished observations). The constitutive cell-specific expression of a suicide gene in CD11c^+^ DCs entailed the development of a myeloproliferative disorder in mice [28].

We have investigated the function of two distinct DC subsets in atherosclerosis and an overview of these studies will be provided in (Table 1).

## 2. CCL17-Expressing DCs Modulate Adaptive Immune Responses in Atherosclerosis

As the CC chemokine CCL17 is exclusively expressed by a myeloid-related mature subset of cDCs [29], we used mice with a targeted replacement of the* Ccl17* gene by the enhanced green fluorescent protein gene (*Egfp, Ccl17* ^E/E^) in order to elucidate the localization and function of CCL17^+^ DCs in atherosclerosis. While we could confirm the presence of numerous CD11c^+^ DCs in atherosclerosis-prone regions of naive mice, these did not express CCL17 under physiological conditions. However, when we placed* Ccl17* ^E/+^ *Apoe* ^−/−^ and* Ccl17* ^E/E^ *Apoe* ^−/−^ mice on a high fat diet, CCL17^+^ DCs could be localized in atherosclerotic lesions in the aortic root, with no differences between genotypes. Moreover, in bone marrow transplantation experiments we similarly evidenced CCL17^+^ DCs in plaques of* Apoe* ^−/−^ mice carrying* Ccl17* ^E/+^ bone marrow [23], indicating that these DCs (or their precursors) are continuously recruited during lesion growth.

Deficiency in CCL17 inhibited atherosclerotic lesion formation at different stages of plaque progression and disease models, that is, diet-induced and spontaneous lesion formation. In* Apoe* ^−/−^ mice deficient in CCL17, atherosclerotic lesions were characterized by a reduction in macrophage and T-cell content [23]. A number of studies have demonstrated that the accumulation of activated T cells within atherosclerotic lesions, for example, triggered in response to MIF, was associated with an enhanced atherosclerotic plaque growth [30, 31]. CCL17 and CCL22 were previously shown to activate the chemokine receptor CCR4 and to attract effector/memory T cells of the Th1 and Th2 subtype but also regulatory T cells (Tregs) [32–34]. In line with previous studies showing that CCL17 enhances CD4^+^ T-cell and Treg recruitment [32–35], CCL17 attracted both CD4^+^ T cells and Tregs to an inflammatory air pouch [23]. In conjunction with other proadhesive molecules expressed within plaques [36], CCL17 expressed by mature DCs in the vessel wall may thus function to recruit T cells to the vessel wall. Indeed, adoptively transferred CD4^+^ T cells homed to aortas of* Ccl17* ^+/+^ *Apoe* ^−/−^ mice but less abundantly to those of* Ccl17* ^E/E^ *Apoe* ^−/−^ mice. In contrast, Treg cell recruitment was minimal and not altered in* Ccl17* ^+/+^ *Apoe* ^−/−^ versus* Ccl17* ^E/E^ *Apoe* ^−/−^ mice. In the contrary, we found an increased accumulation of Tregs in aortas of* Ccl17* ^E/E^ *Apoe* ^−/−^ mice and in LNs of* Ccl17*-deficient mice, indicating that a higher Treg content is not due to their selective or preferential recruitment, which clearly can occur independently of CCL17, but rather due to effects on Treg maintenance. When further investigating this option, we indeed observed an enhanced expansion or increased rate of conversion of Tregs and an increased proliferation of CD4^+^ T cells and Tregs* in vivo *when monitoring the fate of adoptively transferred T cells in* Ccl17*-deficient mice [23]. These data provided first evidence that CCL17^+^ DCs indeed control the maintenance of Tregs. Similarly, an expansion of Tregs has subsequently also been observed in a model of intestinal inflammation in* Ccl17* ^E/E^ mice [37]. Tregs are instrumental for maintaining self-tolerance and preventing uncontrolled inflammation or autoimmune disease [38]. Moreover, Tregs act as powerful inhibitors of atherosclerosis [39, 40]. Our data corroborate that an expansion of Tregs in the absence of CCL17^+^ DCs protects mice from atherosclerotic lesion formation.

It remains to be determined whether immune responses are initiated and sustained in the arterial wall or in secondary lymphatic tissue. Although CCL17^+^ DCs can be localized in close proximity and direct contact to CD4^+^ T cells, and some proliferating CD4^+^ T cells are present within lesions, only few Tregs are detectable in atherosclerotic plaques [23]. Together with the marginal recruitment of Tregs to the inflamed aorta, this implies that CCL17^+^ DCs may be involved in homeostatic mechanisms primarily within lymphoid tissue. Thus, it is likely that the primary site for Treg control is secondary lymphoid tissue, from where Tregs may be recruited to sites of inflammation at low numbers. The increased Treg accumulation in atherosclerotic aortas of* Ccl17*-deficient mice [23], however, further implies that lesional CCL17^+^ DCs may contribute to homeostatic mechanisms at the site of inflammation. Thus, CCL17 expressed in atherosclerotic plaques may also serve to locally constrain Treg maintenance to some extent, thereby propagating inflammation [23].

The underlying pathways engaged by CCL17 that mediate these effects on T cells remain to be conclusively addressed. Although a role of CCR4 in recruiting CD4^+^ T cells and Tregs has been shown* in vitro *[32], we could not confirm a predominant role of this receptor* in vivo*, as outlined above. Moreover, deficiency in* Ccr4* did not phenocopy the effects of CCL17-deficiency* in vivo*, and lesion formation was unaltered in chimeric low density lipoprotein receptor-deficient (*Ldlr* ^−/−^) mice reconstituted with* Ccr4* ^+/+^ versus* Ccr4* ^−/−^ bone marrow [23]. The mechanisms enacted by CCL17 thus do not solely seem to be mediated by CCR4, implying the contribution of other CCL17 receptors or potential heteromer partners.

Downstream of its receptor on T cells, we could gather evidence that CCL17 may interfere with STAT5 phosphorylation. Given the importance of IL-2 in STAT5 activation to sustain Foxp3 expression in Tregs, involving STAT5 binding to a highly conserved STAT-binding site located in the first intron of the* Foxp3* gene [41], interference with STAT5, as seen in T cells interacting with CCL17^+^ DCs [23] may not only constrict T-cell proliferation but may also diminish conversion into Foxp3^+^ Tregs and their peripheral maintenance. Although not experimentally addressed in detail, we speculate that CCL17 may block the mitogen-activated protein kinase pathway, as seen for CCL17-induced interference with Src-kinase- and ERK-phosphorylation, or the PI3K pathway [42–44].

We could thus reveal that CCL17 characterizes a DC-subset that is of paramount importance in the initiation and progression of atherosclerosis. In line with findings detecting CCL17 and high numbers of myeloid DCs in advanced human plaques [16–18], the upregulation of CCL17 transcripts in human carotid endatherectomy specimens as compared to macroscopically healthy arteries [23] underscores the possible clinical relevance of our findings.

## 3. pDCs and Autoimmunity in Atherosclerosis

We have also been interested in another DC subset, namely, pDCs in atherosclerosis, as this DC subset has previously been found in carotid artery plaques [21]. Moreover, reduced circulating pDC counts have been described in patients with coronary artery disease, which may correspond to their enhanced accumulation within atherosclerotic lesions [45]. In line with these studies, we observed an accumulation of pDCs in murine and human atherosclerotic plaques and an increased expression of pDC markers in advanced versus early human atherosclerotic carotid artery lesions [46].

The subset of pDCs is specialized for sensing pathogenic single-stranded nucleic acids in viral and microbial infections via the expression of TLR7 and 9, and to produce large amounts of type I interferons (IFN-*α*/*β*) in infection [47, 48]. Accordingly, also plaque-residing pDCs were shown to respond to type A oligodinucleotides (CpGs), which contain motifs typically found in microbial DNA, with an enhanced IFN-*α* expression, which in turn promote inflammatory TLR4, TNF-*α*, IL-12, and matrix metalloproteinase-9 expression by myeloid DCs and cytolytic T-cell functions within human plaques [20, 21], suggesting proatherogenic functions of this DC subset and a function as inflammatory amplifiers.

In order to scrutinize the role of pDCs in atherosclerosis we employed a specific pDC-depleting antibody. In line with the notion that pDCs exert proinflammatory effects, we could demonstrate that pDCs and their activation critically contribute to early atherosclerotic lesion growth. We and others could show that administration of PDCA1 antibody to deplete pDCs protected from lesion formation in* Apoe* ^−/−^ mice and was associated with reduced macrophage content [46]. Moreover, we could demonstrate that stimulation of* Apoe* ^−/−^ mice with type A CpGs promoted plaque growth in a pDC-dependent manner, as revealed by abrogating CpG-induced lesion formation by the additional depletion of this cell type. Moreover, administration of IFN-*α* enhanced atherosclerotic lesion formation in* Apoe* ^−/−^ mice [46], similar to findings that treatment with another type I interferon, namely, IFN-*β*, accelerated atherosclerotic lesion formation [49]. Notably, pDC-derived type I interferons may accelerate atherosclerotic lesion formation by upregulating chemokines that in turn promote macrophage accumulation in plaques, furthermore emphasizing that plaque residing DC may affect leukocyte recruitment to the vessel wall.

Interestingly, we could demonstrate that oxLDL enhanced the phagocytosis of pDCs and their capacity to prime antigen-specific T-cell responses* in vitro*, while not directly contributing to IFN-*α* responses or costimulatory molecule upregulation [46]. In line, Macritchie et al., showed that antigen-presentation capacities of aortic pDCs were enhanced in* Apoe* ^−/−^ mice in atherosclerosis [50]. Exposure to oxLDL within plaques [2] may thus boost uptake of antigenic complexes by pDCs and their activation in atherosclerosis and indicating that pDC responses to autoantigens may be enhanced in atherosclerosis.

In different autoimmune diseases, such as psoriasis or systemic* lupus erythematosus* (SLE), it has been shown that inert self-DNA fragments that are released, for example, by dying cells, can be bound by the antimicrobial peptide LL37/Cramp to form complexes that can trigger recognition of self-DNA by TLR7/TLR9 by pDCs and the secretion of type I IFNs [48, 51–53]. Antimicrobial Cramp protein can be released from inflammatory cells [54] and neutrophil granules [55]. Also neutrophil extracellular traps (NETs), web-like structures containing self-DNA together with antimicrobial peptides expelled by activated neutrophils during cell death processes termed NETosis, can trigger pDC activation [56–58].

Given the early recruitment of neutrophils in atherosclerosis [30] and an increased expression of LL37 in human atherosclerotic carotid artery plaques compared to normal arteries [59], we further investigated whether antimicrobial peptides can be found in atherosclerotic arteries and whether self-DNA recognition may also occur in atherosclerosis. Indeed, we observed an increased expression of Cramp in atherosclerotic plaques of* Apoe* ^−/−^ mice, which also showed a colocalization with DNA fragments in plaque necrotic core areas and could be detected in the vicinity of segment-nucleated neutrophils within plaques. Moreover, we unprecedentedly also observed the formation of NETs in atherosclerotic carotid arteries [46]. Cramp-self-DNA complexes that could form in atherosclerotic lesions may thus trigger pDC activation also in atherosclerosis. First evidence for a recognition of Cramp-self-DNA complexes in atherosclerosis was indeed derived from our study that showed diminished anti-dsDNA antibody titers in* Ldlr* ^−/−^ mice reconstituted with* Cramp* ^−/−^ versus* Cramp* ^+/+^ bone marrow, together with a protection from atherosclerotic lesion formation, and decreased anti-dsDNA antibody titers in pDC-depleted* Apoe* ^−/−^ mice [46]. These data suggested that Cramp-dependent break-down of tolerance to self-DNA may stimulate pDCs and IFN-*α* production, contributing to the formation of anti-dsDNA antibodies in atherosclerosis. pDC-derived type I IFNs may in addition activate other immune cells, such as cDCs and B cells, to promote autoimmunity [60]. The comparatively low levels of such autoimmune activity (when compared to overt autoimmune disease) may be related to a locally confined pattern of this mechanism.

Notably, anti-dsDNA antibodies may be deposited within plaques, and we detected the presence of immunoglobulin IgG deposits within atherosclerotic arteries [46], in line with previous work [61]. Given that such antibody complexes that contain self-DNA as well as antimicrobial peptides can furthermore trigger pDC activation and IFN-*α* production [52, 57, 58, 62–64], anti-dsDNA antibodies, generated as a consequence of pDC-activation, may contribute to the pathogenic insult in atherosclerosis. Complementing data* in vitro*, serum of* Apoe* ^−/−^ mice containing high levels of anti-dsDNA antibody titers but not serum containing low titers indeed significantly increased IFN-*α* production in pDCs* in vitro* [46]. Increased levels of circulating anti-ds-DNA antibodies in atherosclerotic* Apoe* ^−/−^ mice, as well as in patients with symptomatic and more advanced atherosclerosis, may support a pathogenic role of anti-dsDNA antibodies also in humans [46]. Importantly, elevated anti-nuclear antibody titers were associated with decreased carotid elasticity in young Finns and postulated to participate in the development of early atherosclerosis [65]. Moreover, in an animal model of SLE with enhanced antibody titers against dsDNA, atherosclerotic lesion formation was accelerated [66]. Chronically increased IFN-*α* levels and circulating anti-dsDNA antibody titers in patients with psoriasis and SLE [48] may likewise predispose for an increased risk to develop hyperlipidemic atherosclerosis [67].

The responsiveness of pDCs to viruses or bacterial infections, as epitomized by treatment of* Apoe* ^−/−^ mice with CpGs, may in addition corroborate a link between atherosclerosis and chronic infection burdens and possibly the inflammatory activation of vulnerable plaques in response to acute infections [20].

## 4. Future Challenges and Conclusions

Due to their ability to regulate T-cell responses, we and others have explored DCs as potential therapeutic targets. As a prerequisite, we have contributed to the understanding of the mechanisms and mediators engaged by DCs to drive atherosclerosis. As illustrated by the inhibitory effects of an antibody to CCL17 on atheroprogression in* Apoe* ^−/−^ mice [23], we have shown that DC-derived CCL17 could represent an attractive molecular target, which may be translated into new therapeutics for preventing atheroprogression. Moreover, the specific depletion of pDCs, which at least in mice was shown to be feasible [46], or blocking of its effector cytokine IFN-*α*, may constitute alternative approaches for treating atherosclerosis.

Given the remarkable role of immunity in atherosclerosis, targeting of its cellular constituents holds promises for new therapeutic approaches to ameliorate the disease process. Plaque-residing DCs, as epitomized by CCL17^+^ DCs or pDCs, may function to recruit T cells and to promote local antigen contact and T-cell instruction in the vessel wall [23, 46], although T-cell responses affecting plaque growth may primarily be systemically modulated within lymphoid organs. Further studies will be required to resolve whether immune response are initiated and maintained in the vessel wall or in lymphoid organs. Nevertheless, effects of interfering with T-cell and DC recruitment to the vessel wall could be exploited therapeutically.

The understanding that T and B cells and their immune responses critically control atherosclerosis suggests that atheroprotective vaccination may constitute another approach for disease modulation. Given that antigen-specific immune mechanism could be initiated by both CCL17^+^ DCs and pDCs [23, 46],* ex vivo* loading of DCs with antigen or the targeting of DC receptors for delivery of antigen to (these) specific DC subsets may be explored in atherosclerosis, similar to approaches for treatment of cancer, where specific humoral and cellular responses could be evoked by vaccination strategies and have evolved as viable therapeutic options [68, 69]. Treatment of patients with full-grown lesions that have developed over years may be challenging in the clinical setting. Vaccination studies in mice with established atherosclerosis are warranted as a first experimental approach.

Although CD11c is commonly accepted as a pan-DC marker also for vascular cells [9–12], a clear identification of DCs is still limited by the lack of unambiguous surface markers. In particular, the discrimination of DCs from macrophages that share many markers and functions remains challenging. Recently, the evolutionarily conserved, previously uncharacterized zinc finger transcription factor zDC was identified to be expressed specifically by cDCs and their immediate precursors but not by monocytes or other bone marrow-derived cells and targeted by inserting DTR cDNA into the 3′UTR of the zDC gene (zDC-DTR mice) [70]. Employing zDC-DTR mice may provide a first understanding of the role of true DCs in atherosclerosis.

Aortic CD11c^+^MHCII^+^ DCs have recently been functionally discriminated from CD11c^−^MHCII^+^ macrophages by their low phagocytic activity but strong immune stimulatory capacities [9]. We and others have however shown that the majority of aortic CD11c^+^DCs can furthermore be subdivided into different subpopulations using the surface markers CD11b, F4/80, CD103 [9, 71], and PDCA [46, 50].

It remains to be determined if DCs and their subsets (and vascular macrophages) represent true lineage subsets with distinct transcriptional profiles, as already investigated for other tissues in large gene-expression and regulatory-gene network databanks (Immunological Genome, ImmGen) [72]. Gene expression analysis of these distinct DC subsets should similarly be performed to resolve lineage membership. Furthermore, a detailed functional characterization of individual DC subsets should follow. Moreover, the origin of vascular DC subsets, the mechanisms of their recruitment, and the signaling cues involved in their differentiation remain to be elucidated.

Atherosclerotic vascular disease affects different vascular locations and may involve specific cell-cell-interactions and pathways at different disease stages. It remains to be determined if different locations in the vascular tree show differences in the accumulation of specialized DC subsets, in turn reflecting in differences in the susceptibility to disease development. An example of this is the accumulation of CD103^+^ DCs primarily in the aortic root together with changes in atherosclerotic lesion formation primarily at this site upon loss of the CD103^+^ DC subset [9].

Understanding the origin and function of distinct DC subsets that control atherogenesis, and definition of the molecular mechanisms underlying atherosclerosis will open up new strategies to interfere with or to enhance DC subset differentiation and functions for developing novel therapeutic targets and approaches for the treatment of vascular inflammation and atherosclerosis.

## Figures and Tables

**Table 1:** Atherogenic CCL17^+^ DC and pDC functions in a nutshell.

CCL17^+^ DCs	CD4^+^ T-cell recruitment to atherosclerotic lesions Interference with Treg homeostasis Not exclusively mediated via CCR4; involvement of other receptors?

pDCs	oxLDL induces enhanced T-cell activation capacities Recognition of self-DNA (complexed to antimicrobial peptide Cramp) IFN*α* production, contribution to anti-dsDNA antibody generation
